# Effect of propofol and midazolan on microcirculation of septic shock patients

**DOI:** 10.1186/cc10938

**Published:** 2012-03-20

**Authors:** G Penna, F Fialho, A Japiassu, D Salgado, P Kurtz, G Nobre, M Kalichsztein, N Villela, E Bouskela

**Affiliations:** 1 Casa de Saúde São José, Rio de Janeiro, Brazil; 2 IFF-FIOCRUZ, Rio de Janeiro, Brazil; 3 IPEC-FIOCRUZ, Rio de Janeiro, Brazil; 4 UFRJ, Rio de Janeiro, Brazil; 5 UERJ, Rio de Janeiro, Brazil

## Introduction

Septic shock patients are submitted to many therapeutic strategies, including sedation. It is unknown if different sedative drugs influence microcirculation.

## Methods

We performed a prospective observational study, using sidestream dark-field imaging (SDF), to evaluate sublingual mucosa of septic shock patients admitted to our ICU. SDF was applied in two settings: continuous sedation with propofol and with midazolan. We repeated each examination after an interval of 30 minutes. Eight fields (videos) were analyzed during propofol and midazolan infusion. Two videos were obtained from each side of the tongue. The Bispectral index was monitored along with the Richmond Agitation Sedation Scale: the dose of both sedatives was titered to maintain light sedation. All demographic and severity of illness data were collected. Vasopressor agents were maintained to a mean arterial pressure of 70 mmHg and the cardiac index was kept stable through the protocol study.

## Results

We included 15 patients; APACHE II score was (median) 17.5 points and SOFA score 9 points. The Bispectral index was lower in the midazolan group (43 vs. 48.5 points, *P *= 0.005), although RASS was the same for both groups. Large-vessel perfusion was similar for both groups. The small perfusion vessel proportion was significantly reduced with propofol (92 vs. 96.3%, *P *= 0.003). The microvascular flow index was also lower during propofol infusion (MFI - 2.4 vs. 2.7, *P *= 0.002). We observed a higher heterogeneity index when patients were sedated with propofol (0.4 vs. 0.19, *P *= 0.01).

## Conclusion

Propofol reduces small-vessel perfusion and increases the heterogeneity of circulation in the sublingual mucosa, when compared with the use of midazolan in septic shock patients.

